# Interkingdom interactions on the denture surface: Implications for oral hygiene

**DOI:** 10.1016/j.bioflm.2019.100002

**Published:** 2019-06-15

**Authors:** Christopher Delaney, Lindsay E. O'Donnell, Ryan Kean, Leighann Sherry, Jason L. Brown, Gareth Calvert, Christopher J. Nile, Laura Cross, David J. Bradshaw, Bernd W. Brandt, Douglas Robertson, Gordon Ramage

**Affiliations:** a Oral Sciences Research Group, Glasgow Dental School, School of Medicine, Dentistry and Nursing, College of Medicine, Veterinary and Life Sciences, University of Glasgow, 378 Sauchiehall Street, Glasgow, G2 3JZ, UK; b School of Life Sciences, College of Medicine, Veterinary and Life Sciences, University of Glasgow, University Avenue, Glasgow, G12 8QQ, UK; c Department of Biological and Biomedical Sciences, School of Health and Life Sciences, Glasgow Caledonian University, Glasgow, UK; d Oral Health Research and Development, GlaxoSmithKline, St Georges Avenue, Weybridge, Surrey, UK; e Department of Preventive Dentistry, Academic Centre for Dentistry Amsterdam, University of Amsterdam and Vrije Universiteit Amsterdam, Amsterdam, the Netherlands

**Keywords:** Microbiome, Candida, Denture, Oral hygiene

## Abstract

**Background:**

Evidence to support the role of *Candida* species in oral disease is limited. Often considered a commensal, this opportunistic yeast has been shown to play a role in denture related disease, though whether it is an active participant or innocent bystander remains to be determined. This study sought to understand the role of *Candida* species alongside the bacterial microbiome in a denture patient cohort, exploring how the microbiology of the denture was affected by oral hygiene practices.

**Materials and methods:**

In vitro denture cleansing studies were performed on a complex 9-species interkingdom denture biofilm model, with quantitative assessment of retained bacterial and fungal viable bioburdens. Patient hygiene measures were also collected from 131 patients, including OHIP, frequency of denture cleansing, oral hygiene measure and patient demographics. The bacterial microbiome was analysed from each patient, alongside quantitative PCR assessment of ITS (fungal) and 16S (bacterial) bioburden from denture, mucosa and intact dentition.

**Results:**

It was shown that following in vitro *denture* cleansing *C. albicans* were unresponsive to treatment, whereas bacterial biofilms could repopulate 100-fold, but were susceptible to subsequent treatment. Within the patient cohort, oral hygiene did not impact candidal or bacterial composition, nor diversity. The levels of *Candida* did not significantly influence the bacterial microbiome, though an observed gradient was suggestive of a microbial composition change in response to *Candida* load, indicating interkingdom interaction rather than an oral hygiene effect. Indeed, correlation analysis was able to show significant correlations between *Candida* species and key genera (*Lactobacillus, Scardovia, Fusobacterium*).

**Conclusions:**

Overall, this study has shown that the denture microbiome/mycobiome is relatively resilient to oral hygiene challenges, but that *Candida* species have potential interactions with key oral genera. These interactions may have a bearing on shaping community structure and a shift from health to disease when the opportunity arises.

## 1 Background

Denture microbiology has historically focused on the role of the fungi Candida albicans and other members of the genus. Clinical studies demonstrate clear associations between high quantities of Candida spp. and Newton's classification levels of disease severity [[1], [2], [3]]. Indeed, it has been further suggested that the individual strains of C. albicans and its capacity to form biofilms also plays a role in disease outcome in denture-related disease [4], a characteristic trait that has also been shown to be true in other systemic diseases [[5], [6], [7]]. Moreover, it has been shown that strain specificity also has a bearing on denture cleansing capacity, with those individuals harbouring more prolific biofilm properties negatively responding to treatment [8]. Taken together, these studies support the notion of a simple mono-species oral infection, and justify the disproportionate focus on Candida spp., particularly related to assessing denture oral hygiene strategies in vitro and in vivo [9,10].

Dentures are nevertheless also bathed in the salivary microbiome, a microbial soup interacting on the surface of the denture acrylic. The resultant denture plaque biofilms are complex, and are often represented by dense, mixed interkingdom communities [11]. The quantities of bacteria and fungi found residing upon the pores and varied denture topography are vast, yet remain relatively unexplored [12,13]. The first molecular-based microbial denture studies revealed a complex bacterial microbiota with potentially cariogenic, periodontopathic and malodorous capacities [[14], [15], [16]]. Moreover, in mixed dentition where there is the presence of natural teeth within a partially edentulous patient, differential ecology is observed that may have a bearing in the progression of oral disease [17]. Despite these studies providing greater insights into the complexity of oral bacterial biofilms ecology, they fail to account for the involvement of Candida spp. within the community. We know these fungi are prone to contribute to a less diverse biofilm [12,18], which can also play a leading role in driving denture-related stomatitis. With this in mind and based on our greater understanding of the importance of Interkingdom interactions within the denture biofilm, investigating both the bacterial and fungal role during the development and testing of new oral hygiene products seems prudent. Our group were the first to recently undertake a randomised double blinded control trial to assess the importance of frequency of denture cleaning [19], where both bacteria and fungi were quantified as primary outcome measures. This study statistically demonstrated the benefit of frequent (daily) cleansing regimens compared to intermittent regimens. Nevertheless, a key limitation of the study was the failure to employ next generation sequencing (NGS) techniques to fully assess the microbial composition of patients under different cleaning regimens. Given the importance of both bacteria and fungi within the denture environment, in addition to the impact of oral hygiene on denture stomatitis, the aim of this study was to assess the contribution of oral hygiene measures and the relevance of Candida spp. to the denture microbiomes of edentulous patients, with the ultimate aim of improving denture antimicrobial strategies.

## 2 Methods

### 2.1 In vitro denture cleansing study

A denture plaque cleansing study and quantitative analysis of remaining viable cells was performed as previously described [20]. The intention behind this study was to investigate the impact of frequent denture cleansing regimens on bacterial and fungal retention on denture acrylic, with an aim to understand how complex interkingdom biofilms respond to oral hygiene measures. Specifically, this involved a combined chemical and mechanical brushing cleansing technique, employed sequentially over a 7-day treatment period.

Briefly, laboratory strains were used to create a polymicrobial denture plaque biofilm model based on the most dominant genera/species identified from our recent denture microbiome study [12,19]. Polymethylmethacrylate (PMMA) discs were manufactured as described as previous [21], providing the physical substrates on which biofilms were formed. The biofilms included Streptococcus mitis NCTC 12261, Streptococcus intermedius ATCC 27335, Streptococcus oralis ATCC 35037, C. albicans 3153A, Actinomyces naeslundii ATCC 19039, Veillonella dispar ATCC 27335, Rothia dentocariosa DSMZ 43762, Lactobacillus casei DSMZ 20011 and Lactobacillus zeae DSMZ 20178. Initially, S. mitis, *S. intermedius*, S. oralis and C. albicans were grown and standardised in artificial saliva (AS) to 1 × 107 cells/mL. The AS was comprised of porcine stomach mucins (0.25% w/v), sodium chloride (0.35 w/v), potassium chloride (0.02 w/v), calcium chloride dihydrate (0.02 w/v), yeast extract (0.2 w/v), lab lemco powder (0.1 w/v), proteose peptone (0.5 w/v) in ddH2O (Sigma, Poole, UK). Urea was then added to independently to a final concentration of 0.05% (v/v). The standardised microbes were added to each well of a 24 well plate (Corning Inc, New York, USA) containing 13 mm2 PMMA discs (Chaperlin and Jacobs Ltd, Southend-on-Sea, UK) and incubated aerobically at 37 °C for 24 h. Next, standardised (1 × 107 cells/mL) A. naeslundii, V. dispar, R. dentocariosa, L. casei and L. zeae were added to the preformed 24-h biofilm and incubated at 37 °C in 5% CO2 conditions for a further 4 days. Spent biofilm supernatants were removed and replaced with fresh artificial saliva daily.

The treatment regimen was a daily treatment (days 1–7) of a 3 min soaking with a denture cleanser (Polident®3-min denture cleanser; GSK Consumer Healthcare, Weybridge, UK) followed by brushing with filter sterilised hard water (HW) at 375 ppm CaCO3. For analyses, sample biofilms were assessed pre- and post-treatment. Following each treatment, PMMA discs were incubated in Dey-Engley neutralising broth (Sigma-Aldrich, Gillingham, UK) for 15 min. PMMA discs were then sonicated in phosphate buffered saline (PBS [Sigma-Aldrich, Gillingham, UK]) at 35 kHz for 10 min to remove the biomass, as previously described [10]. For quantitative analysis, live/dead quantitative PCR (qPCR) were performed [20]. Live/dead PCR was performed using 16S and 18S bacterial and fungal specific primers, and quantified using appropriate bacterial and fungal standard curves [22]. Data distribution, graph production and statistical analysis were performed using GraphPad Prism (version 5; La Jolla, CA, USA). After assessing whether data conformed to a normal distribution, One-way Analysis of Variance (ANOVA) and t tests were used to investigate significant differences between independent groups of data that approximated to a Gaussian distribution. A Bonferroni correction was applied to the p value to account for multiple comparisons of the data.

### 2.2 Study participants and sample collection

Denture wearing patients attending the University of Glasgow Dental School and Hospital were enrolled in the study, as described previously [12]. Written informed consent was obtained from all participants. Ethical approval for the study was granted by the West of Scotland Research Ethics Service (12/WS/0121). Clinical assessments were carried out by six experienced dentists working in the prosthodontic department of the University of Glasgow Dental Hospital and School. All prosthodontists received personal training from DR (senior clinical lecturer in restorative dentistry and principal investigator) in order to standardise the assessment of the clinical disease (inflammation), denture retention, stability, occlusion and cleanliness. Oral/denture hygiene was graded after training and discussion, as either excellent, good or poor. Patients were required to complete a questionnaire covering a number of aspects concerning their oral hygiene and oral hygiene routine. Within the questionnaire were questions relating to the Oral Health Impact Profile-14 (OHIP). The OHIP is an overall score given by 14 items on a patient questionnaire. These questions encompass oral functional process reported by the patient as well as the psychological impact and assess the overall quality of life of a denture wearer within this study [23]. All examiners were trained but no formal calibration calculations were carried out. The patient demographic and clinical examination data was recorded on a standardised data collection sheet.

Ethylene oxide sterilised swabs (Fisher Scientific, Loughborough, UK) were used to take samples from the denture surfaces in contact with the palatal mucosa and the palatal mucosal surface covered by the dentures. Samples were collected and processed, as previously described [12]. In total, samples from 131 patients were collected, which included 131 denture swabs, 131 mucosal swabs and 79 dental plaque samples. However, during DNA extraction process not all samples had sufficient DNA, and therefore only DNA from 108 denture samples, 87 mucosal samples and 63 dental samples remained for sequencing, collectively all these samples originated from 123 patients. In parallel, dentures removed from the patients’ mouth were placed in sterile bags (Fisher Scientific) containing 50 ml PBS (Sigma-Aldrich, Dorset, UK). Adherent denture plaque was then removed by sonication (Ultrawave, Cardiff, UK) for 5 min, as previously described [12]. Bacterial and fungal loads were quantified by qPCR using 16S and ITS primers, as described previously [12,24].

### 2.3 DNA extraction and Illumina sequencing

All samples were prepared for DNA isolation as previously described [12], using a combination of chemical and mechanical lysis. Briefly, the plaque samples and the swabs samples were suspended in TE buffer before being transferred to a well within a plate containing 0.25 ml of lysis buffer (AGOWA mag Mini DNA Isolation Kit, AGOWA, Berlin, Germany), 0.3 g zirconium beads (diameter, 0.1 mm; Biospec Products, Bartlesville, OK, USA) and 0.2 ml phenol. The samples were then homogenized by with a Mini-beadbeater (Biospec Products) before DNA was extracted using the AGOWA mag Mini DNA isolation kit.

The amplicons were sequenced in paired end mode on a MiSeq sequencing system (Illumina, Eindhoven, the Netherlands) with the v2 kit (Illumina) [25,26]. The paired-end reads where quality-filtered and processed into an OTU table with taxonomic annotation, as described previously [12]. The sequencing data are available at the NCBI with bioproject ID: PRJNA324548 (http://www.ncbi.nlm.nih.gov/bioproject/324548).

### 2.4 Study design and statistical analyses

OTU data was pre-processed according to our previous study [12]. Statistical analysis was performed within R on the OTU and taxonomic tables created, as described previously. Additionally, meta table data from clinical parameters and other in vitro analysis including CFEs from qPCR were used for analysis in this study. Candida load was measured as the proportion of ITS gene over the 16S gene abundance and this value was normalised by a log10 transformation. Candida load was further categorised as high, medium or low by separation of data into 3 quantiles. Community analysis was performed using both the Simpson and Shannon alpha-diversity indexes. These indexes were calculated with the use of the R package phyloseq [27]. Nonmetric Distance Scaling (NMDS) plots of community data were performed using Bray-Curtis distances on community data represented as OTUs. Additionally, principal coordinates analysis (PCoA) using the Weighted Unifrac distance measure was used. To measure OTUs that significantly differed between conditions, we used the R package DESeq2 [28], with a false discovery rate FDR adjusted p-value of 0.01 and log2 fold-change cut-off of 1.5. OTUs that were differing in abundance in one variable compared to another were displayed as an MA plot were the log2 transformed fold change is plot against the mean abundance and those OTUs that met the above criteria in DESeq2 are displayed. Additionally, Pearson correlation analysis was performed on normalised count data and analysis of variance (ANOVA) were performed within R. Correlations of different genera were performed against different oral hygiene variables and Candida load. OTUs with less than 50 reads were also trimmed from the dataset when performing correlation analysis.

## 3 Results

### 3.1 Influence of denture hygiene on in vitro biofilms

**Fig. 1:**
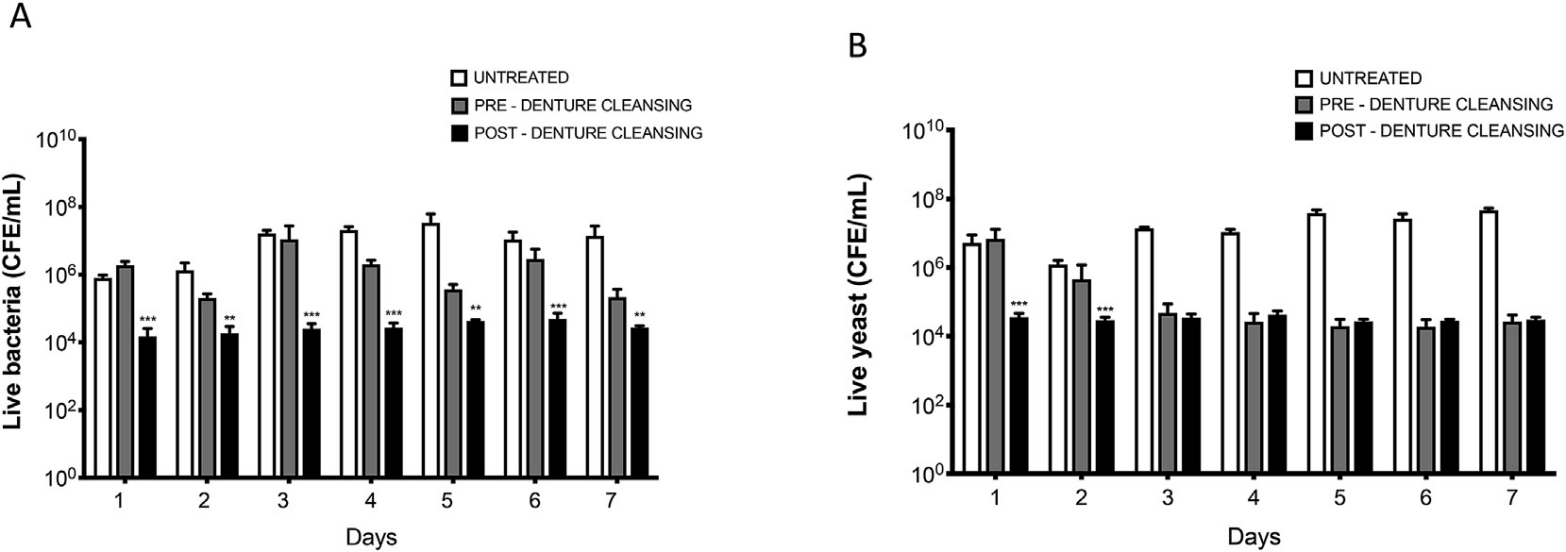
**Quantitative live assessment of bacterial and *Candida* load following *in vitro* denture hygiene.** Nine species denture biofilms were grown on PMMA sections, followed by daily denture cleanser and brushing. Sonicate samples were taken pre- and post-treatment, and an untreated positive control included. Live cell numbers were determined by qPCR or PMA treated samples (live cells) of **(A)** bacteria, and **(B)***Candida*. Data was analysed by ANOVA with a Bonferroni correction (**p ​< ​0.01, ***p ​< ​0.001).

In vitro denture biofilms were exposed to a combination of chemical and mechanical denture cleansing sequentially over a 7-day period. Live cell analysis was carried out by qPCR using the 16S and 18S rDNA primers. A control arm with no intervention was included for comparison. Fig. 1 shows that denture cleansing was able to significantly reduce the viable bacterial colony forming equivalents (CFEs) by at least 1 log10 when comparing pre-treatment to post-treatment samples on each day of analysis (p < 0.01). However, when the Candida were quantified, we could not detect any significant difference in cell numbers within the treatment group pre- and post-denture cleansing, despite highly significant differences between the treatment arm and control group from days 3–7 (p < 0.001). Supplementary Fig. 1 shows that total bacteria and Candida CFEs are higher in all control and treatment arms, suggesting dead cells make up a considerable component of the dead biofilm. Taken together, these data indicate that bacteria within the biofilm are more sensitive to denture cleansing than Candida, alternatively, bacterial numbers during regrowth are able to supress the ability of retained Candida cells to repopulate. Irrespective, significant numbers of Candida and bacteria are retained despite intense and frequent treatment regimens, suggesting some co-operative protection or tolerance within an interkingdom biofilm.

### 3.2 Patient demographics

131 patients were recruited to this study, of which the primary demographics of these patients are shown in Table 1. The average patient age was 70.2 ± 11.5 years (min: 33, max: 95) with an average denture age of 4.5 ± 5.1 years (min: 0.2 max: 40). In terms of gender, females represented the majority of the population at 64.9%, with males contributing only 35.1%. After clinical diagnoses, 62.6% of participants were found to have healthy oral mucosa and the remaining 37.4% were diagnosed with DS.

**Table 1:** Patient denture hygiene demographics.

	Healthy	Disease
N	82 (62.6%)	49 (37.4%)
Male	26 (31.7%)	20 (40.8%)
Female	56 (68.3%)	29 (59.2%)
Mean Age	71.6	68.2
Median Age	72	70
Mean Age of Denture	4.5	4.4
Complete dentures	61 (74.4%)	28 (57.1%)
Partial dentures	21 (25.6%)	21 (42.9%)

Denture/Oral Hygiene		
Excellent	16 (19.5%)	5 (10.2%)
Good	49 (59.8%)	25 (51.0%)
Poor	17 (20.7%)	19 (38.8%)

Denture cleaning		
once/day	24 (30.0%)	18 (37.5%)
≥ twice/day	56 (70.0%)	30 (62.5%)

Sleeping with denture		
No	43 (52.4%)	15 (30.6%)
Yes	39 (47.6%)	34 (69.4%)

Patients were classed as having excellent, good or poor denture/oral hygiene; Clinicians classed participants as having excellent (16%), good (56.5%) or poor (27.5%) oral hygiene. When separated into healthy and diseased groups, 20.7% and 38.8%, respectively, were classed as having poor denture hygiene. Denture cleaning varied among the cohort, however, the majority reported cleaning their dentures either once or twice per day. Forty-two (32.1%) participants reported cleaning their denture once per day, whereas 86 (65.6%) cleaned theirs twice per day. Going to sleep whilst wearing a denture is a habit that was commonplace amongst study participants, as 73 (55.7%) of the total patients reported that they sleep with their denture in situ (Table 1).

### 3.3 Influence of denture hygiene on the denture associated Candida

**Fig. 2:**
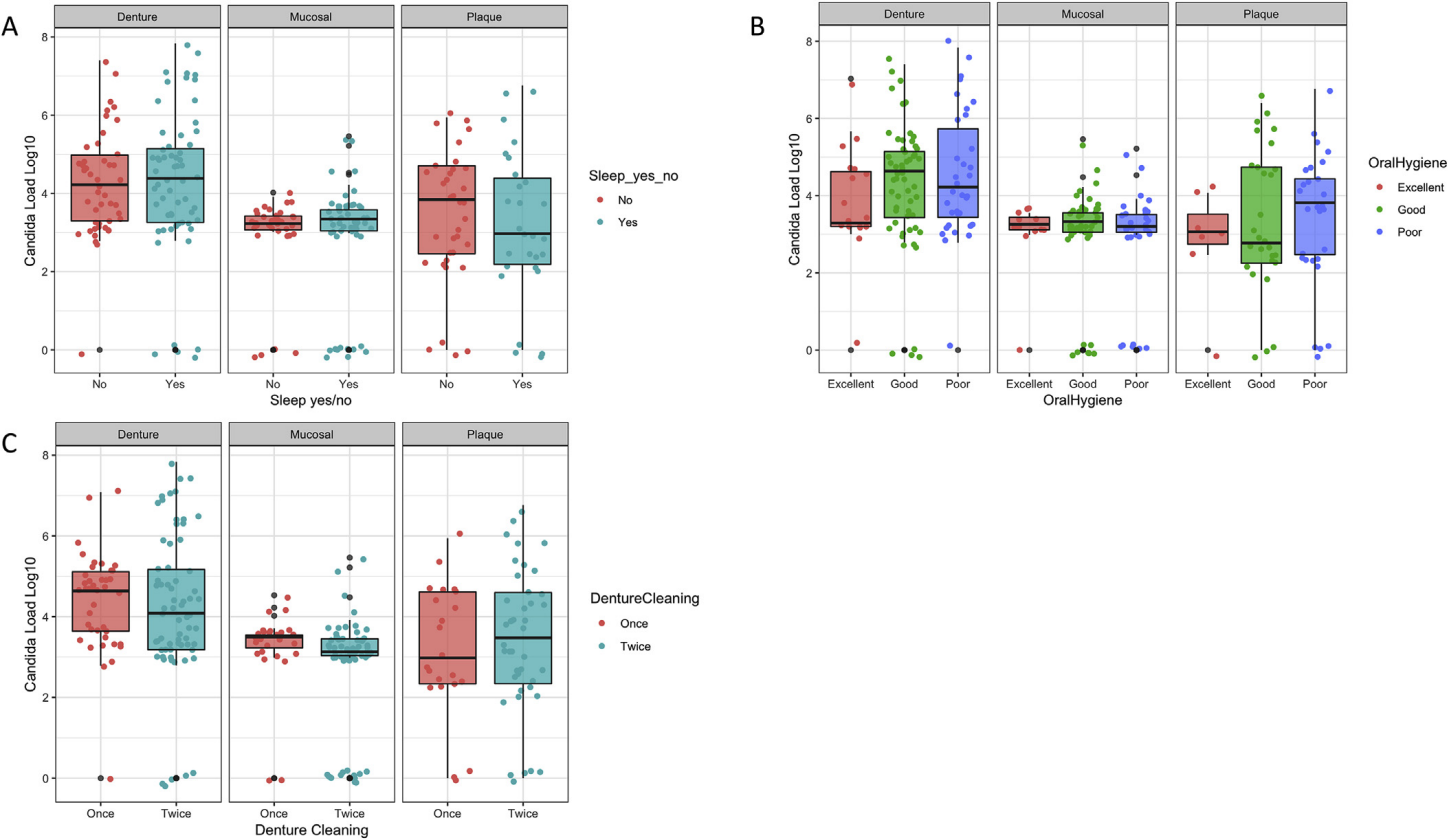
**Candida load between hygiene variables and oral sites**. Total *Candida* load represented as log_10_ within the denture, mucosal and plaque microbiota, as assessed by qPCR. The *Candida* load is compared at each of these sites between those who slept in their dentures and those who did not (A) those with poor, good or excellent oral hygiene (B) and individuals who cleaned there dentures once or less than once a day and those who cleaned their dentures twice or more times a day (C).

The influence of Candida load on dentures was compared across a number of oral and denture hygiene practices (Fig. 2). In regard to sleeping with the denture in situ, no statistical significance was observed between those who did and those who did not with respect to Candida load (Fig. 2A). Whether the denture wearer had good, poor or excellent oral hygiene similarly appeared to have no effect on the Candida load (Fig. 2B), nor did cleaning once or twice per day have an impact on Candida burden (Fig. 2C). These data suggest that Candida species are not influenced by oral hygiene measures in vivo.

### 3.4 Influence of denture hygiene on the oral microbiome

PCoA ordination plots utilising the weighted UniFrac distance measure were used to evaluate the difference in diversity between the different oral and denture hygiene practices. When using abundance and phylogenic distances (Weighted UniFrac) to compare hygiene status, denture cleaning and sleeping with the denture in situ we observed no patterns in diversity between the different conditions (Supplementary Fig. 2). The Shannon and Simpson alpha-diversity were implemented to compare the diversity within the different oral hygiene groups. When using the alpha-diversity scores Shannon and Simpson to compare between conditions, no significant differences were observed (Supplementary Figs. 3–5). The diversity was not significantly affected by the hygiene status, denture cleaning frequency or whether an individual sleep in their denture within this cohort (Supplementary Figs. 3–5). These data suggest that bacterial species, composition and diversity are not influenced by oral hygiene measures in vivo.

**Fig. 3:**
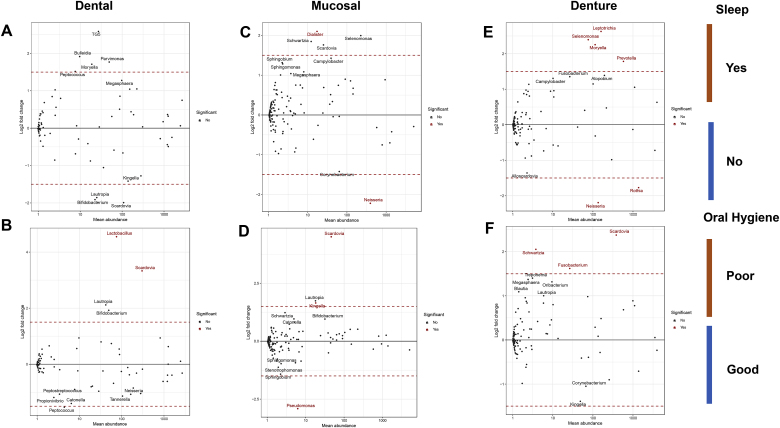
**Measure of taxa in significantly higher abundance between patient variables.** MA plots, which are Log2 fold change plotted the against mean abundance, depict taxa that are in differing levels of abundance between patient variables. The patient variables are; whether or not the patient sleeps with their denture in and their overall hygiene rating. The level of each genus between the two patient variables in the Dental **(A, B)**, Mucosal **(D, C)** and Denture **(E, F)** microbiomes are indicated by each individual dot. The top ten taxa are labelled with their genus name and significant taxa, with an FDR adjusted p value < 0.01, are indicated in red.

Despite their being no overarching consequence on the diversity and richness of the oral community between different hygiene measures, some individual changes in species abundance were observed with respect to oral hygiene status and those who did and did not leave their dentures in situ whilst sleeping (Fig. 3). No significant changes in abundance of species were observed in the dental microbiome (Fig. 3A). However, the mucosal microbiome (Fig. 3C) had 1 genus significantly represented (Dialaster) in those who slept with denture in situ. The denture microbiome (Fig. 3E) had a number of genera (Leptotrichia, Selenomonas, Moryella, Prevotella) in significantly higher abundance in those who slept with their denture in situ.

Within the dental, mucosal and denture microbiome a number of species were in significantly higher proportion in those with poor oral hygiene (Fig. 3). The bacterial genus Scardovia was in significantly higher abundance in all three microbiome sites (Fig. 3B, D and F) in those who had poor oral hygiene. Those with poor oral hygiene also had higher levels of Fusobacterium and Schwartzia in the denture microbiome (Fig. 3F), and increased levels of Lactobacillus within their dental microbiome (Fig. 3B). These data indicate that specific genera of bacteria are influenced by oral hygiene practices.

### 3.5 Influence of Candida load on hygiene and microbial communities

**Fig. 4:**
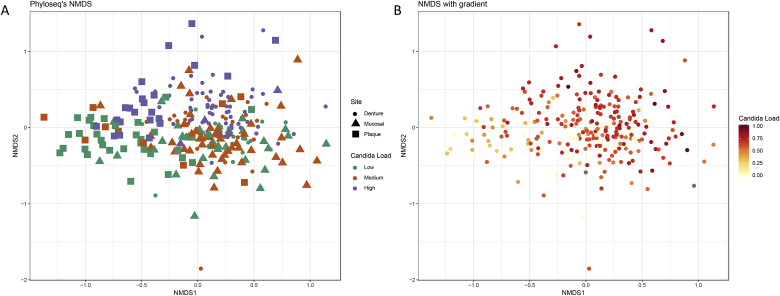
**Non-metric dimensional scaling of OTU data based upon the Bray-Curtis distance measure.** Community data is indicated by oral site using shapes and *Candida* load is indicated as either low, medium or high by colour **(A)**, community data is represented as a gradient of *Candida* load (ITS/16S) **(B)**. When comparing diversity between the low, medium and high *Candida* loads we observed that the overall abundance of different OTUs slightly decreases. Similarly, when comparing the diversity between low, medium and high *Candida* load with the *Shannon* and *Simpson* diversity measures there is a small reduction in diversity, from the low to the medium and high *Candida* load (Fig. 5). No significance in diversity was found using any of the three measures between low, medium and high *Candida* load.

We next compared the levels of Candida, which were ascertained by qPCR and converted to colony forming equivalent (CFE) counts and normalised to bacterial CFEs using amplification of ITS and 16S region [12,24]. The Candida load was then compared between the three denture hygiene metrics denture cleaning frequency, oral hygiene and whether the denture was left in situ whilst sleeping. When comparing the overall Candida load between those who did and those who did not sleep in their denture, we observed that there was no discernible difference (Fig. 4A). This was found to be true at each of the oral microbiome sites denture, mucosal and plaque. We also found that there was no significant difference in the Candida load when testing with ANOVA. Similarly, when comparing the Candida load between those with poor, good or excellent oral hygiene at each of the three sites there was no significant difference in the overall Candida burden (Fig. 4B). The frequency of denture cleansing appeared to have no visible effect on the Candida load on denture, mucosal or plaque samples and was statistically insignificant when using an ANOVA to compare the two cohorts.

**Fig. 5:**
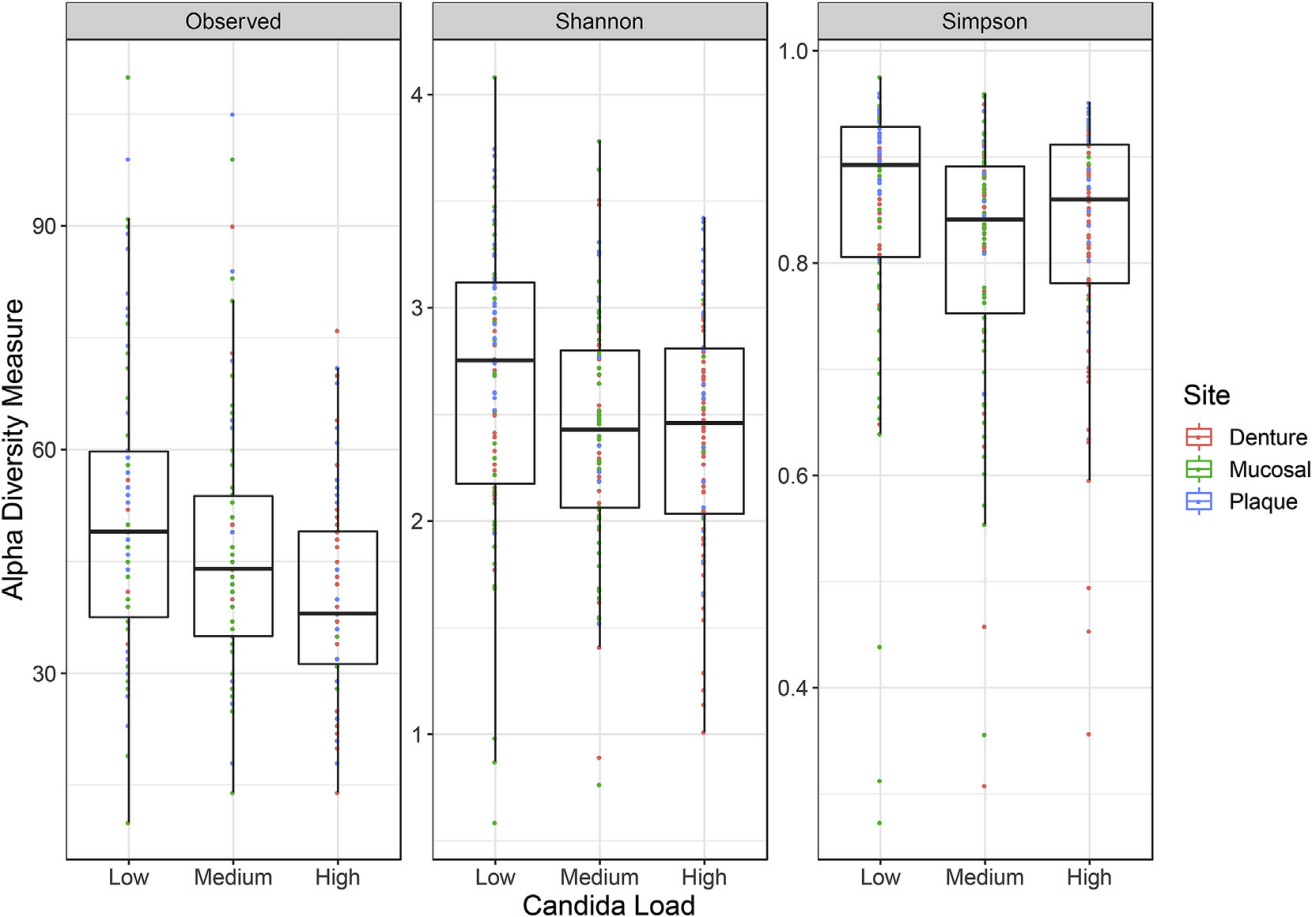
**Microbiome diversity measures between *Candida* load low, medium and high**. Diversity between patients with low, medium or high *Candida* load were measured for all patients. The diversity metrics included are the *Observed* number of OTUs, *Shannon* and *Simpson* diversity indexes. Analysis of variance was performed between patient variables for each site on each of the diversity measures and was only reported if p < 0.05. None of the variables fulfilled these criteria.

In addition to the impact of oral hygiene we considered the relationship between the levels of Candida and the composition and diversity of the bacterial community. Diversity as measured using Bray-Curtis was used to elucidate differences in bacterial diversity due to Candida load. Candida was compared between low, medium and high loads. The NMDs plots do not show distinct separation of clusters relating to the low, medium and high Candida loads due to little dissimilarity between the samples (Fig. 4A). Although there are no distinct clusters of bacterial communities between Candida loads, a continuous gradient in the ordination of the points can be observed in relation to the abundance of Candida from low to high, as shown by the gradient of Candida load (Fig. 4B).

**Fig. 6:**
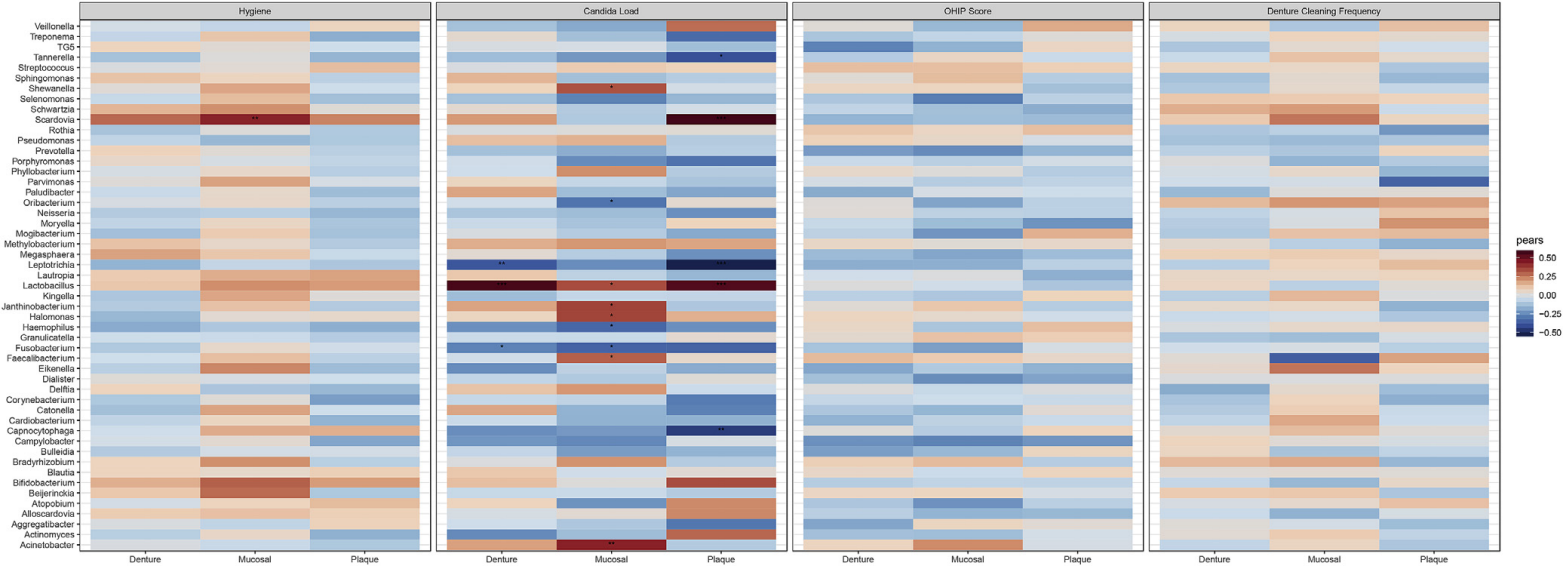
**Correlations of OHIP, oral hygiene variables and *Candida* load with the abundance of a specific genera on the mucosal, denture and dental surface. Heatmap depicting the specific correlations between clinically relevant patient meta-data.** This meta data is comprised of physician measured (Hygiene) and patient reported data (Denture Cleaning Frequency and OHIP) as well as the relative *Candida* load (ITS/16S). The *p-*values were corrected using the Benjamini Hochberg false discovery rate. Significance is indicated by corrected p ​< ​0.05*, p ​< ​0.01**, p ​< ​0.001***.

Finally, hygiene measures including the overall hygiene score, the OHIP score and denture cleaning frequency were all tested for correlations with genera of bacteria. OHIP score and denture cleaning frequency did not correlate significantly with any genera of bacteria, as illustrated in Fig. 6. The bacterial genus Scardovia was positively correlated with oral hygiene, implying an increased level of Scardovia within the mucosal microbiome as oral hygiene measures diminishes. A higher Candida load was correlated with a significantly higher level of genera including Lactobacillus at all three oral sites. Within the mucosal microbiome the abundance of the genera Acineobacter, Faecalibacterium, Janthinobacterium, Halomonas and Shewentalla is positively correlated with an increased Candida load. Within the plaque microbiome Scardovia is positively associated with increased levels of Candida. Conversely, specific genera such as Leptotrichia are negatively associated with Candida in both the plaque and denture microbiome. Other significant negative correlations include Tannerella (plaque), Captnocytphaga (plaque), Fusobacterium (denture & mucosal), Oribacterium (mucosal) and Haemophilus (mucosal). These data suggest that Candida species have a subtle influence on the bacterial microbiome in denture patients and can significantly influence specific genera. Oral hygiene measures had less influence on bacterial genera comparatively.

## 4 Discussion

As the elderly population expands, the number of denture wearers will coincidently rise. In the UK population, approximately 20% wear removable dentures, with 70% of UK adults older than 75 years old wearing dentures [29]. There are a variety of factors that potentially influence the onset and severity of denture-related disease, which in addition of denture cleanliness and pH of denture plaque, includes denture base material, age of denture, continuous denture wearing, denture trauma, smoking, dietary factors and immune status [30,31]. Dentures can influence oral health status, particularly in relation to the oral microbiome. Soft tissue inflammation results from persistent exposure to microorganisms, a characteristic of denture stomatitis [4]. Numerous bacterial and fungal species frequently adhere to the denture surface and form a biofilm amongst cracks and crevices of acrylic substrates [29]. Here, we report for the first time the relationship between denture hygiene practices, the oral microbiome and fungi. The data presented demonstrates the importance and resilience of Candida species alongside bacteria within an interkingdom biofilm amongst the denture wearing population.

Currently, there are a limited number of denture related microbiome studies available in the literature [12,13,16]. We have utilised the microbiome data from our clinical trial to develop a representative model of denture plaque based on the most dominant represented genera [19], an iteration of a previous in vitro denture model [20]. Our in vitro denture cleansing study revealed that the bacterial component of a 9 species biofilm was able to significantly regrow 100-fold compared to the yeast population over 24 h post treatment. This effect most likely supressed the ability of the yeast cells to repopulate. However, at the same time the bacterial biofilm was sensitive to subsequent chemical and mechanical disruption, unlike the retained C. albicans component. The study revealed that despite clear differentiation from an untreated biofilm, the levels of live C. albicans retained on the acrylic surface did not change when challenged by a denture cleansing regime. These data suggested that a baseline level of C. albicans was retained and supported bacterial regrowth in amongst dead cells. The use of a more sensitive molecular assay was a primary reason for observing this effect, one that would otherwise be missed using standard microbiological plating [10]. As previously discussed, qPCR is more sensitive technique compared to microbial plating being able to amplify hard to culture organisms and also lower levels of organisms [20]. However, it has been limited by its inability to distinguish between viable and non-viable cells. The Live/Dead qPCR is able to overcome this limitation of qPCR. Giving it advantage of being higher sensitivity and able to quantify viable and non-viable cells. Collectively, these observations suggest a level of tolerance, or persistence, that has evolved over the period of treatment. The mechanisms underpinning this may relate to enhanced extracellular matrix production, changes in cellular physiology, activation of heat shock proteins, changes to cell wall, or interkingdom co-cooperation [[32], [33], [34]]. Regardless, further studies are required to fully elucidate how these resilient populations of Candida and mixed bacterial biofilms survive and repopulate the denture surface.

The results from this analysis prompted us to revisit microbiome data we had obtained in our previous microbiome analysis, where primary outcome measures were focused on disease subtypes [12]. In this study design we had collected and collated patient-related data, including oral hygiene information. Given our in vitro analysis outcomes, we hypothesised that Candida species present in the clinical samples may also be more resilient to oral hygiene interventions. One caveat to the analysis is that the study design is cross-sectional in nature. Our clinical data supported the notion of biofilm insensitivity, however even though Candida levels were unimpacted the bacterial microbiome was also shown to be uninfluenced by routine oral hygiene practices (composition and diversity). Interestingly, a difference in the abundance of specific genera was observed, both on the denture and the mucosa. In those who slept with their denture in situ, differences in the prevalence of the genera Leptotrichia, Selenomonas, Moryella, Prevotella and Dialaster were observed. Little is known about these bacteria, all of which are Gram-negative, anaerobic rods, in regard to their role in denture health. Interestingly, only Prevotella has been shown to be more highly represented in denture stomatitis sufferers in microbiome studies [8,13]. Moreover, poor oral hygiene resulted in Scardovia and Lactobacillus at significantly higher abundance on the dental surface, along with Fusobacterium and Schwartzia in the denture microbiome. Although they are only small community shifts, these genera of bacteria suggest a subtle dysbiosis correlating with reduced oral hygiene standards.

To establish the impact of Candida load on any microbiome change and how these were impacted by oral hygiene measures, we normalised Candida levels based on bacterial load according to established methods [24]. Denture cleansing frequency and the other measures appeared to have no visible effect on the Candida load on denture, mucosal or plaque samples, a result mirroring our own in vitro observation. Indeed, there are no measurable changes in diversity indices across the different Candida loads. One of the caveats of the study design is the cross-sectional nature and lack of power, thus non-significant results between these variables are not necessarily absence of effect, but rather a result of not achieving the optimum sample size required. Despite their not being distinct global changes in composition of the bacterial microbiomes influenced by Candida load, a gradient of low to high Candida load can be observed to influence the bacterial composition relative to abundance. This suggests again that subtle changes to the microbial composition are reflected by changes in abundance of Candida rather than the oral hygiene intervention.

Our final analysis was implemented to discern specific changes in the bacterial composition. It involved correlation analysis at the genus level and the influence of a range of variables, including overall hygiene score, the OHIP score and denture cleaning frequency. This approach enabled us to observe clear positive and negative associations with different oral sites, including the denture surface, and pick out significant correlations. We deemed this an important tactic, as the breadth and depth of literature is now beginning to demonstrate the importance of interkingdom relationships in oral health [11]. Neither OHIP score and denture cleaning frequency were shown to correlate significantly with any genera of bacteria, though Candida load and oral hygiene did reveal significant associations. As has been described elsewhere, a higher Candida load correlated with a significantly higher level of Lactobacillus species in other mucosal sites, which have been typically found to have an antagonistic relationship with Candida [35,36]. Lactobacillus spp. have been shown to inhibit adhesion of Candida and a reduction in biofilm formation. Despite this reported antagonism it is interesting that Lactobacillus spp. are positively associated with Candida in the oral cavity. It is unclear whether the antagonism is species or site dependant, and whether there is an interkingdom synergism between Candida and oral Lactobacillus spp.. Further studies are required to fully elucidate the interactions between these oral microbes, such as been described for C. glabrata and Lactobacillus spp., where the CgHog1 pathway has been shown to protect this Candida species during their interactions [37]. Moreover, the bacterial genus Scardovia was positively correlated with oral hygiene. Conversely, specific genera such as Leptotrichia and Fusobacterium are negatively associated with Candida on the denture microbiome. Given that we now understand that mechanisms of adherence between Candida and bacteria such *Staphylococcus aureus* and oral streptococci, which utilise agglutinin-like sequence adhesins (ALS3) [32,38], or Porphyromonas gingivalis which uses InlJ, an internalin protein family, to interact with the same ALS3 adhesin [39], then it is unsurprising that we are able to tease out specific interactions. These analytic approaches, while not hypothesis driven per se, will help pinpoint the bacterial genera we should consider when designing and developing new biofilm models of microbial pathogenesis. Moreover, understanding the important elements of polymicrobial interkingdom interaction, no matter how subtle, could provide useful direction in the development of novel antibiofilm strategies. The concept of the mycofilm [32], where bacteria utilise fungal species as a scaffold to support their own biofilm, is a prime reason we ought to consider C. albicans as the real keystone oral microorganism [40]. Targeting this may be crucial in generating a wider *anti*-biofilm effect.

In summary, this study has been the first to specifically investigate the relationship between denture hygiene, the oral microbiome, and the influence of Candida species in denture wearers. The findings from this study suggest that maintaining good denture hygiene and hygiene practices do not appear to have a strong influence in altering the microbiome but taken positively this indicates a stable microbial population. Candida species appear to tolerate denture cleansing treatments and persist, which makes them influential within complex interkingdom biofilm populations. Therefore, future studies in oral microbiology and beyond should pay closer consideration to the mycobiome and the influence it can have on the bacterial microbiome. The rationale design of therapeutic interventions should be mindful of the difficulties in managing Candida biofilms.

## Appendix A Supplementary data

BIOFLM-D BIOFLM-D
