# Supplementary material for: Interkingdom interactions on the denture surface: Implications for oral hygiene
Source: Biofilm. 2019 Jun 15;1:100002. doi: 10.1016/j.bioflm.2019.100002 (PMC7067236; doi:10.1016/j.bioflm.2019.100002)

## Slide 1
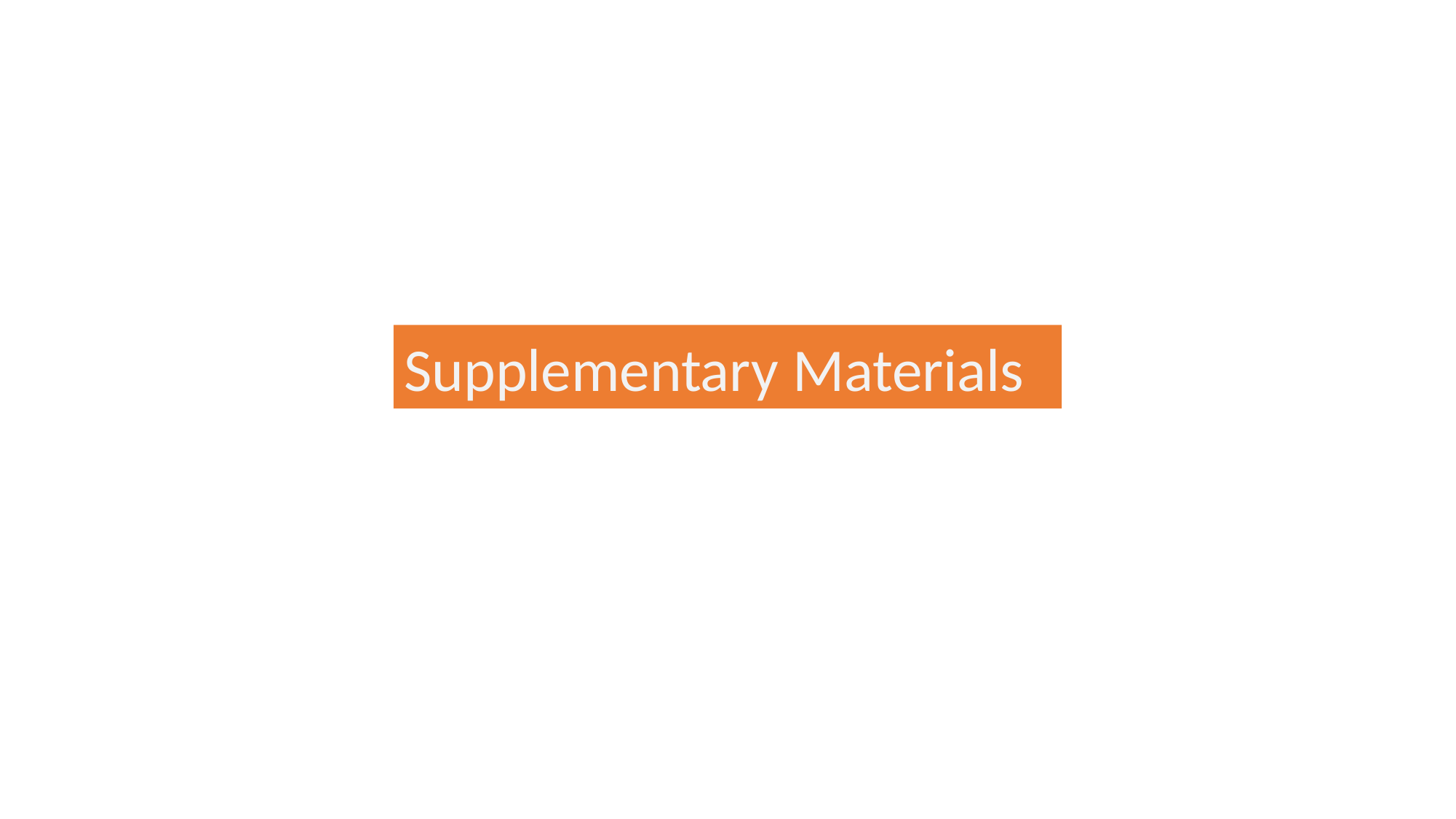

Supplementary Materials

## Slide 2
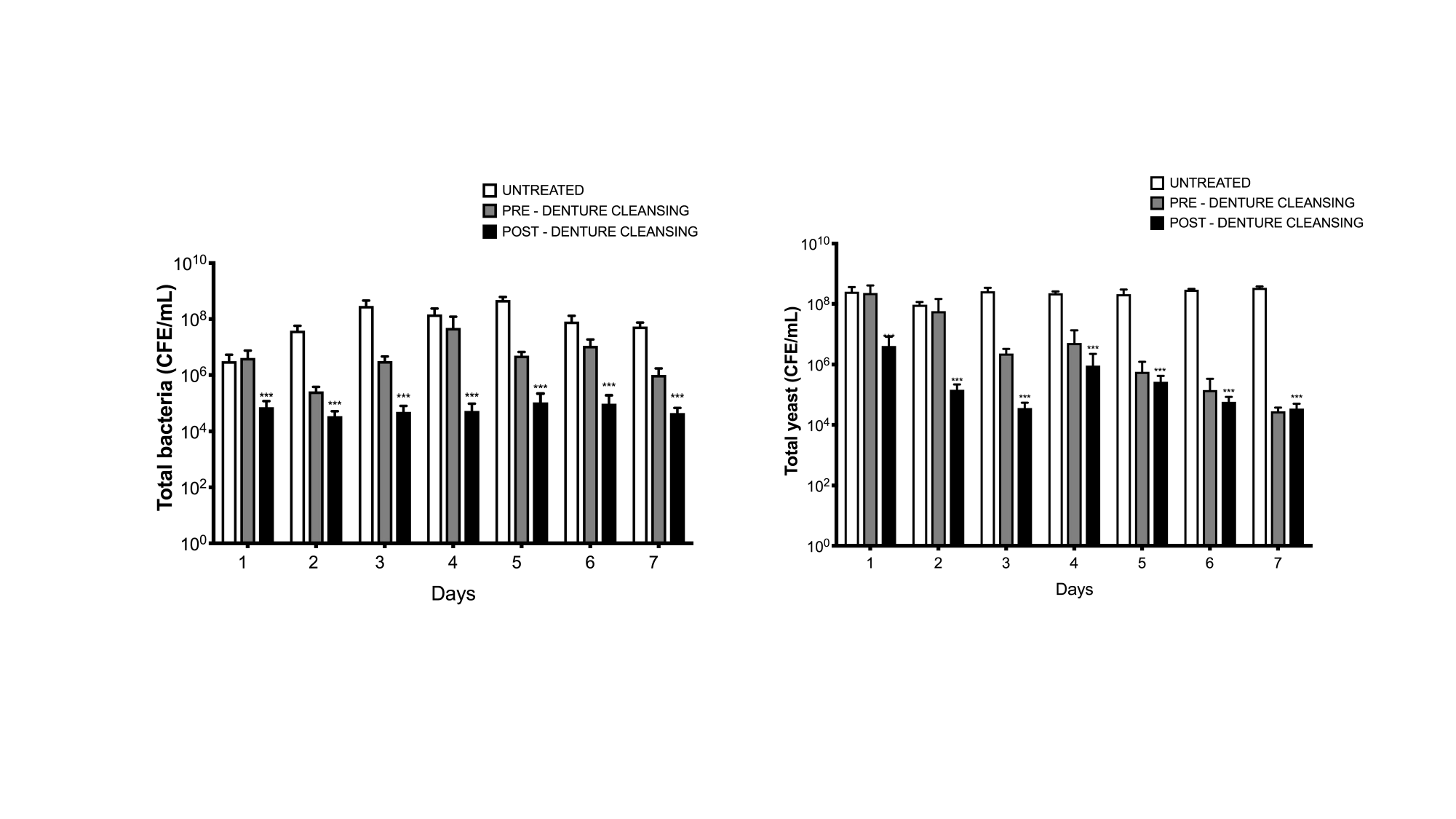

## Slide 3
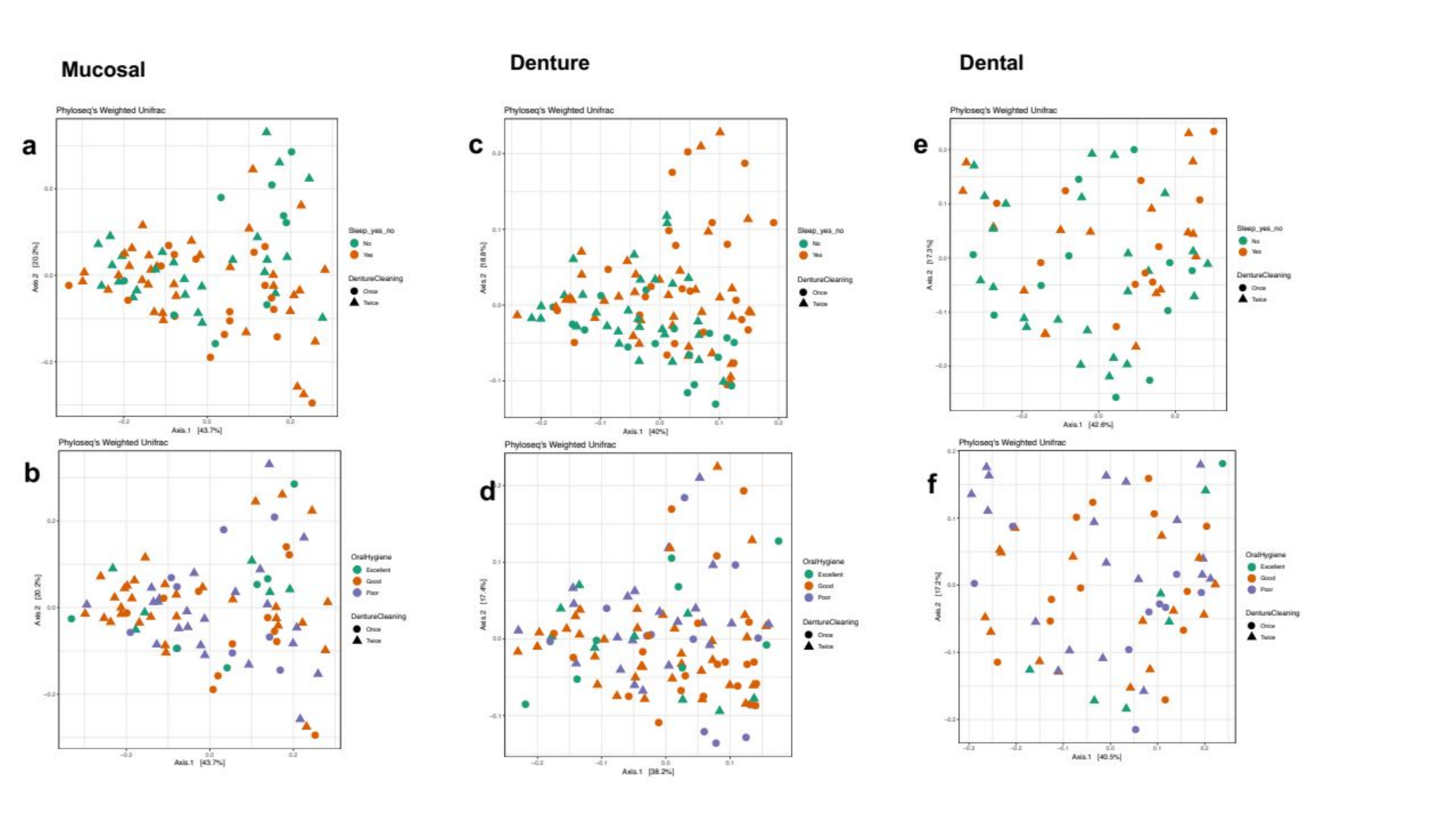

## Slide 4
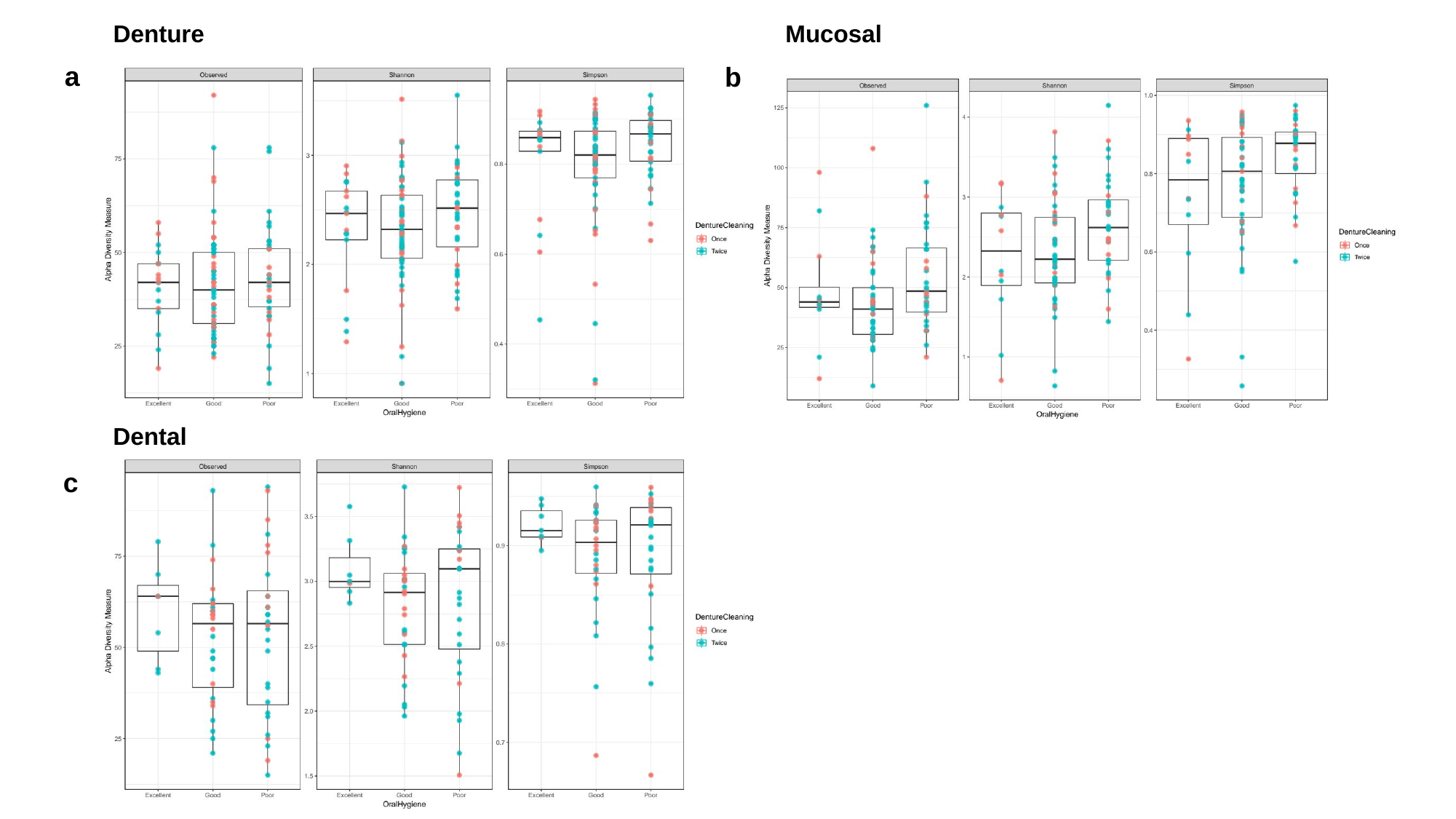

Denture
Mucosal
a
b
b
Dental
c

## Slide 5
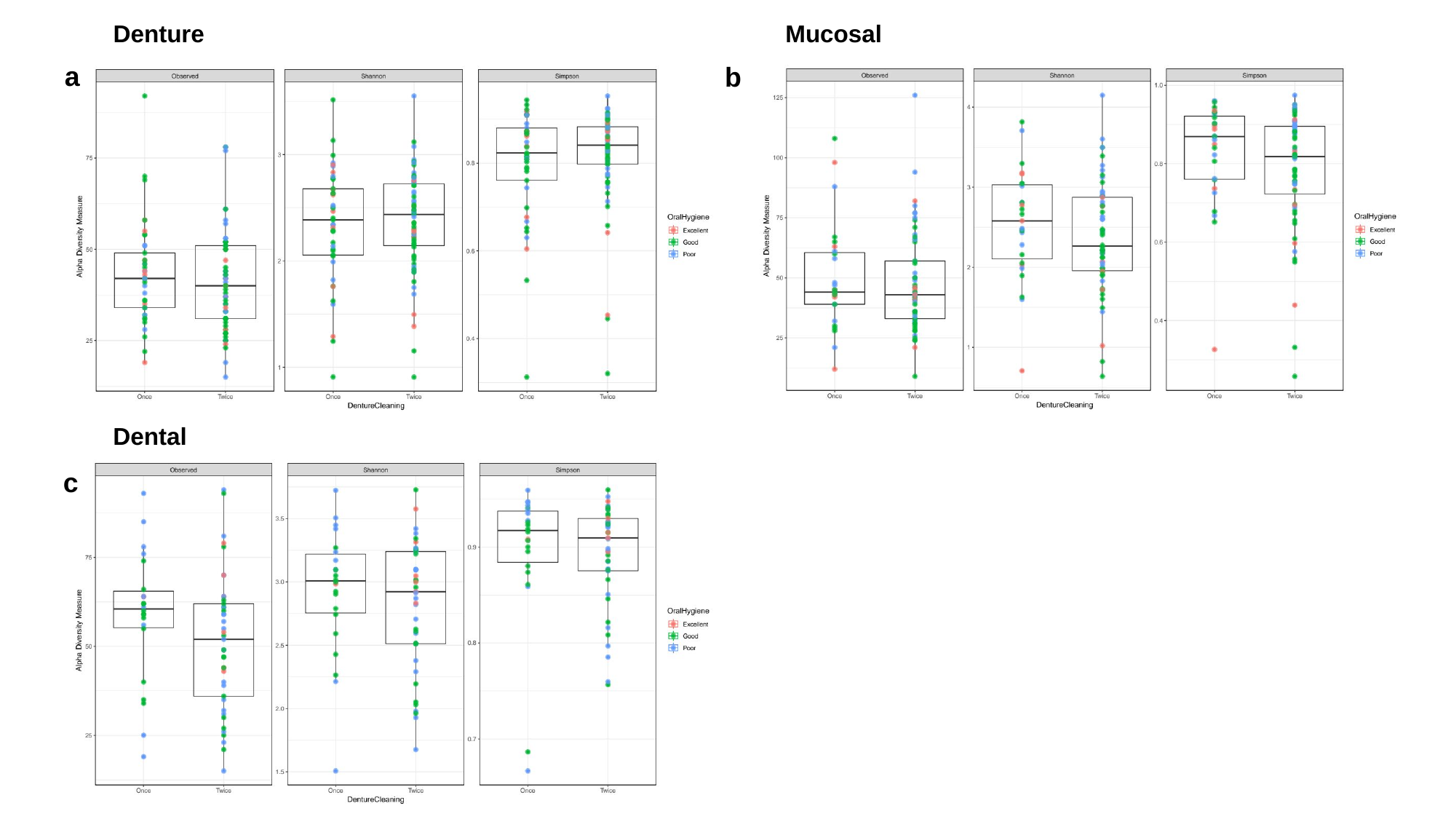

Denture
Mucosal
a
b
Dental
c

## Slide 6
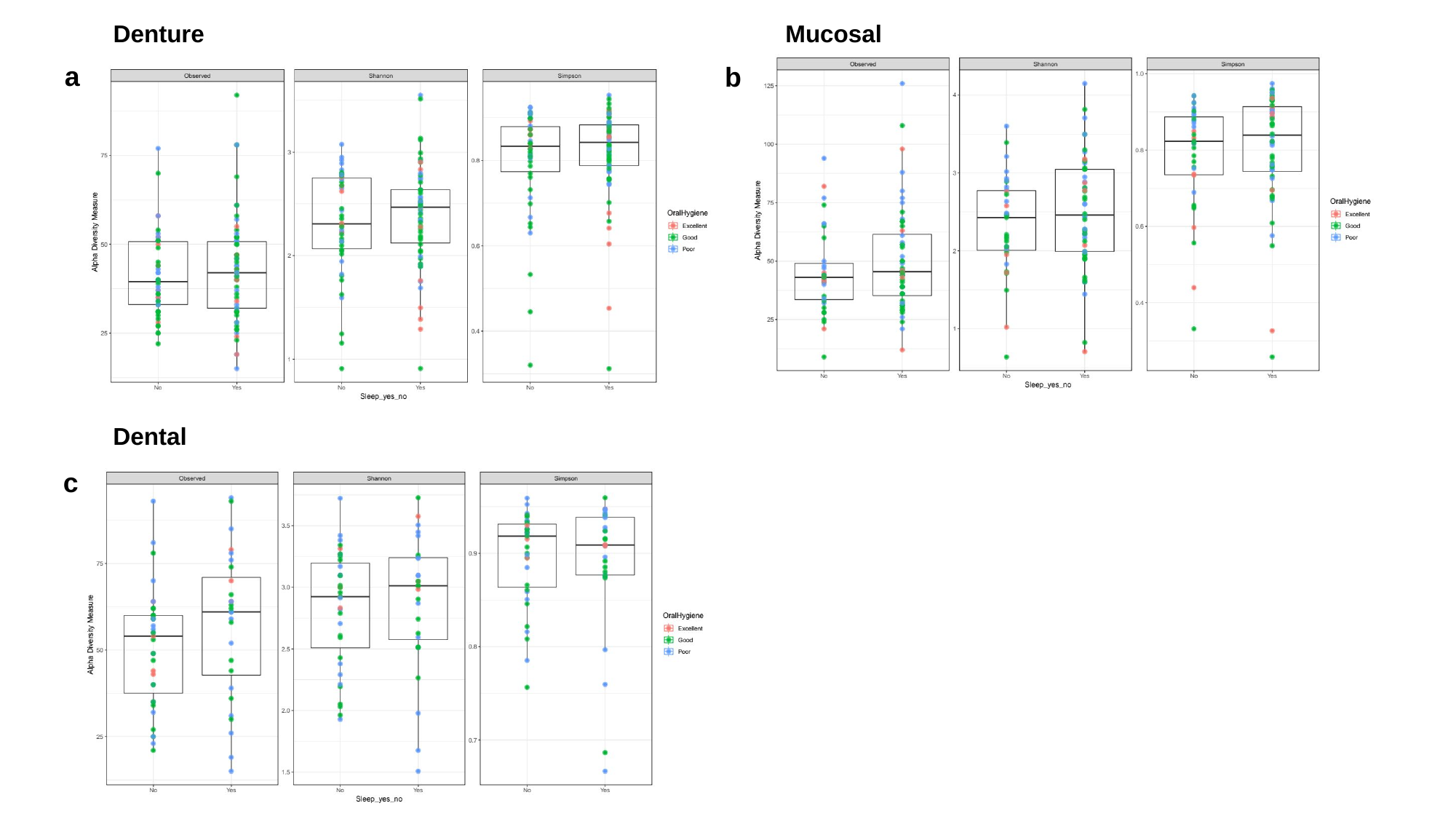

Denture
Mucosal
a
b
Dental
c

## Slide 7
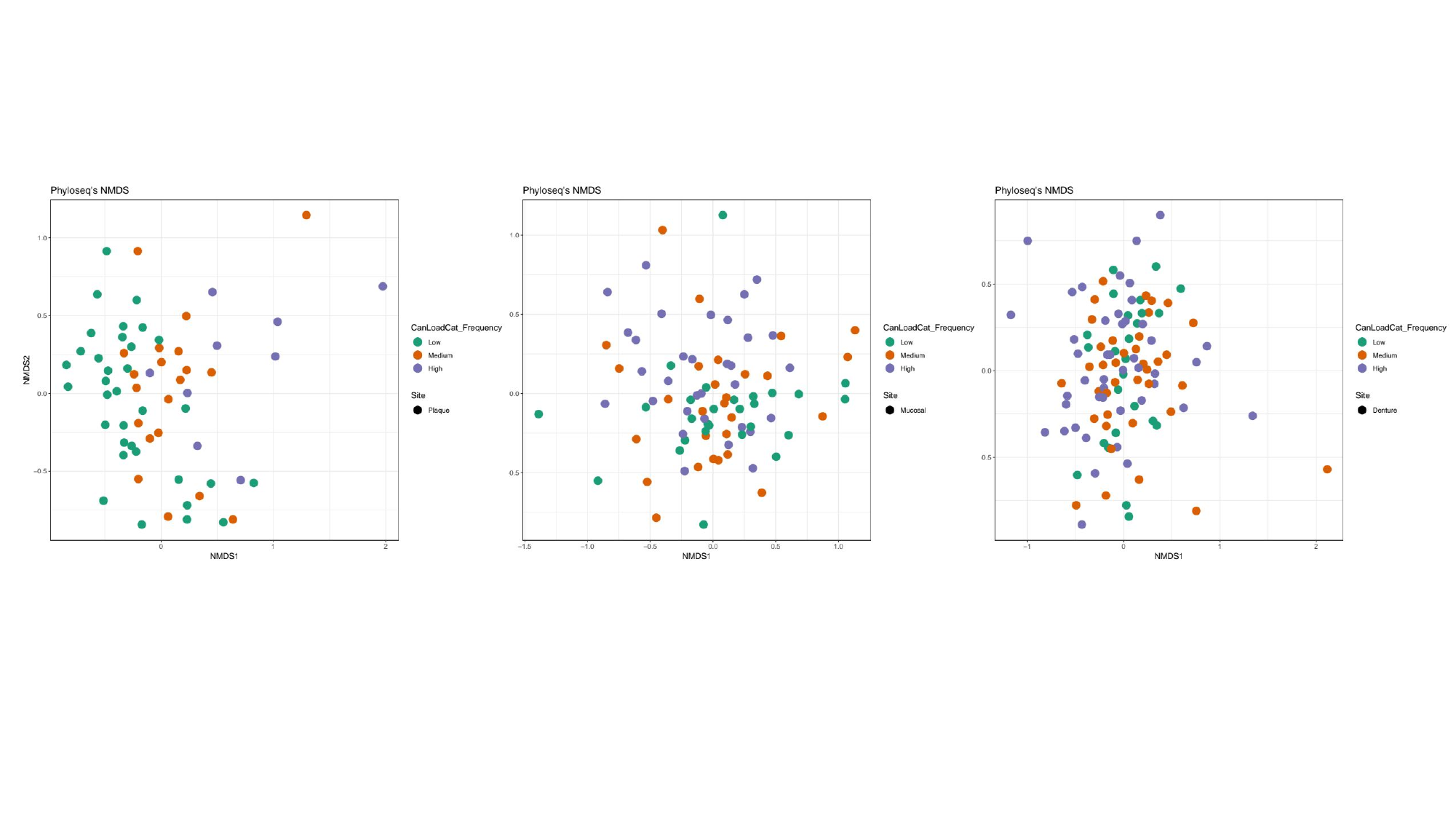

Supplement: BIOFLM-D [file mmc1.pptx]
